# Mechanisms of oxidative stress-induced sperm dysfunction

**DOI:** 10.3389/fendo.2025.1520835

**Published:** 2025-02-05

**Authors:** Yutao Wang, Xun Fu, Hongjun Li

**Affiliations:** Department of Urology, Peking Union Medical Collage Hospital, Beijing, China

**Keywords:** oxidative stress, sperm dysfunction, reactive oxygen species, DNA fragmentation and male infertility, male infertility

## Abstract

Oxidative stress plays a pivotal role in male infertility by impairing sperm function through various molecular mechanisms. This review explores the impact of excessive reactive oxygen species (ROS) on spermatozoa, particularly focusing on lipid peroxidation, DNA fragmentation, and protein oxidation. Lipid peroxidation damages sperm membranes, reducing fluidity and motility. ROS-induced DNA fragmentation compromises genetic integrity, potentially leading to infertility and adverse offspring outcomes. Protein oxidation alters key structural proteins, impairing sperm motility and the ability to fertilize an egg. The primary sources of oxidative stress in sperm include leukocyte activity, mitochondrial dysfunction, and environmental factors such as smoking and pollution. Despite the presence of natural antioxidant defenses, spermatozoa are particularly vulnerable due to limited repair mechanisms. The review highlights the importance of early intervention through antioxidant therapies and lifestyle changes to mitigate the detrimental effects of oxidative stress on male fertility. Further research is essential to enhance therapeutic approaches and improve reproductive outcomes.

## 1 Introduction

Male infertility is a significant global health concern, affecting approximately 8-12% of couples of childbearing age (1–3). Of these, male factor infertility contributes to nearly 50% of cases, underscoring the need for a deeper understanding of its underlying causes. Infertility in men is often associated with abnormalities in sperm quality, including reduced sperm count, motility, and morphology (4, 5). Among the various etiological factors contributing to male infertility, oxidative stress has emerged as a major player, profoundly impacting sperm function and overall reproductive outcomes (6–8). This review aims to explore the critical role of oxidative stress in male infertility, with a focus on the molecular mechanisms through which oxidative stress induces sperm dysfunction.

Male infertility specifically refers to any condition in which a male’s reproductive system impairs the ability to achieve a successful pregnancy. The causes of male infertility are multifaceted and include genetic, environmental, lifestyle, and physiological factors (9–11). While genetic abnormalities, such as chromosomal defects or mutations, play a role in some cases, environmental and lifestyle factors, such as smoking, alcohol consumption, and exposure to environmental toxins, are also significant contributors. Among the physiological factors, oxidative stress is a key determinant of sperm dysfunction (12, 13). Sperm cells are particularly vulnerable to oxidative stress due to their unique characteristics, including high polyunsaturated fatty acid (PUFA) content in their membranes, limited cytoplasmic volume, and minimal antioxidant defenses (14–16). These factors, combined with the fact that spermatozoa cannot repair their DNA as effectively as somatic cells, make them highly susceptible to oxidative damage.

Oxidative stress occurs when there is an imbalance between the production of ROS and the body’s ability to neutralize or detoxify these harmful molecules using its antioxidant defenses. ROS are highly reactive and include free radicals like superoxide anion (O_2_^−^) and hydroxyl radical (•OH), as well as non-radical molecules such as hydrogen peroxide (H_2_O_2_) (17–19). Although low levels of ROS are essential for normal physiological processes, such as sperm capacitation, hyperactivation, and the acrosome reaction, excessive ROS production can be harmful to sperm cells, causing oxidative stress. This imbalance negatively affects sperm function and overall fertility (20–22). In the context of male reproductive health, oxidative stress is a well-established cause of sperm dysfunction. Excessive ROS production damages the sperm membrane, proteins, and DNA, impairing sperm motility, viability, and the ability to fertilize an oocyte. Furthermore, oxidative stress is linked to DNA fragmentation, which has been associated with a higher incidence of infertility, poor pregnancy outcomes, and increased risk of genetic abnormalities in offspring (23, 24). The significance of oxidative stress in male infertility lies not only in its direct impact on sperm quality but also in its potential to induce long-term reproductive and genetic consequences.

Under physiological conditions, a small amount of ROS is essential for sperm functions, including the initiation of capacitation, a process required for sperm to acquire the ability to fertilize an egg. Capacitation involves the hyperactivation of sperm motility and the preparation for the acrosome reaction, a critical step in penetrating the oocyte. ROS participate in this process by modulating signal transduction pathways that promote tyrosine phosphorylation, a key event in sperm activation (25–27). The sperm membrane, rich in PUFAs, is particularly susceptible to lipid peroxidation, a chain reaction that leads to the formation of toxic byproducts such as malondialdehyde (MDA) and 4-hydroxynonenal (4-HNE) (28, 29). These byproducts further compromise the sperm membrane’s integrity, affecting its fluidity and permeability, which are essential for maintaining sperm motility and enabling fusion with the oocyte during fertilization.

In addition to lipid peroxidation, oxidative stress induces protein oxidation, which affects the function of key proteins involved in sperm motility and structure (30–32). For example, oxidative modification of actin and tubulin, the cytoskeletal proteins that maintain the shape and motility of sperm, can lead to a loss of motility and abnormal morphology. Protein oxidation also affects enzymes that regulate sperm metabolism and energy production, further compromising sperm viability. Sperm DNA is highly compacted and protected by protamines, which replace histones during spermatogenesis to ensure tight packaging of the paternal genome. However, the DNA within sperm cells is not completely immune to oxidative damage. ROS can induce single-strand and double-strand breaks in sperm DNA, as well as the formation of oxidative base lesions such as 8-hydroxy-2’-deoxyguanosine (8-OHdG) (33, 34). DNA fragmentation resulting from oxidative stress has been strongly correlated with male infertility and poor reproductive outcomes, including recurrent pregnancy loss and an increased risk of congenital anomalies in offspring.

Oxidative stress in spermatozoa can originate from both internal and external sources. Internally, sperm mitochondria are a major source of ROS. The accumulation of mitochondrial ROS not only impairs ATP production, which is essential for sperm motility but also damages mitochondrial DNA, which lacks the robust repair mechanisms present in nuclear DNA. Mitochondrial dysfunction, therefore, contributes to both reduced motility and compromised sperm DNA integrity (35–37). Externally, leukocytes found in seminal plasma are another major source of ROS. Infections or inflammation in the male reproductive system can activate these leukocytes, causing them to produce high levels of ROS as part of the immune response. Although this process helps eliminate pathogens, the excessive ROS produced near sperm cells can result in oxidative damage, impairing sperm function. Leukocytospermia, a condition characterized by elevated levels of leukocytes in semen, has been associated with increased ROS levels and a higher incidence of sperm DNA fragmentation (38, 39). For example, smoking introduces numerous toxic substances, including free radicals, into the body, leading to increased oxidative damage to sperm DNA, proteins, and lipids.

Given the central role of oxidative stress in male infertility, this review aims to provide a detailed exploration of the molecular mechanisms by which oxidative stress induces sperm dysfunction. Each of these mechanisms will be examined in the context of their impact on sperm function and fertility outcomes. Additionally, the review will discuss the primary sources of oxidative stress in spermatozoa, including mitochondrial dysfunction, leukocyte activation, and environmental/lifestyle factors. Understanding the molecular underpinnings of oxidative stress-induced sperm damage is essential for developing effective therapeutic interventions. By elucidating these mechanisms, this review seeks to highlight potential targets for antioxidant therapies and lifestyle modifications that could mitigate oxidative stress and improve reproductive outcomes in men with infertility. Furthermore, the review will address current gaps in research and propose future directions for studying oxidative stress and its role in male reproductive health.

## 2 Overview of oxidative stress and sperm function

**Figure 1:**
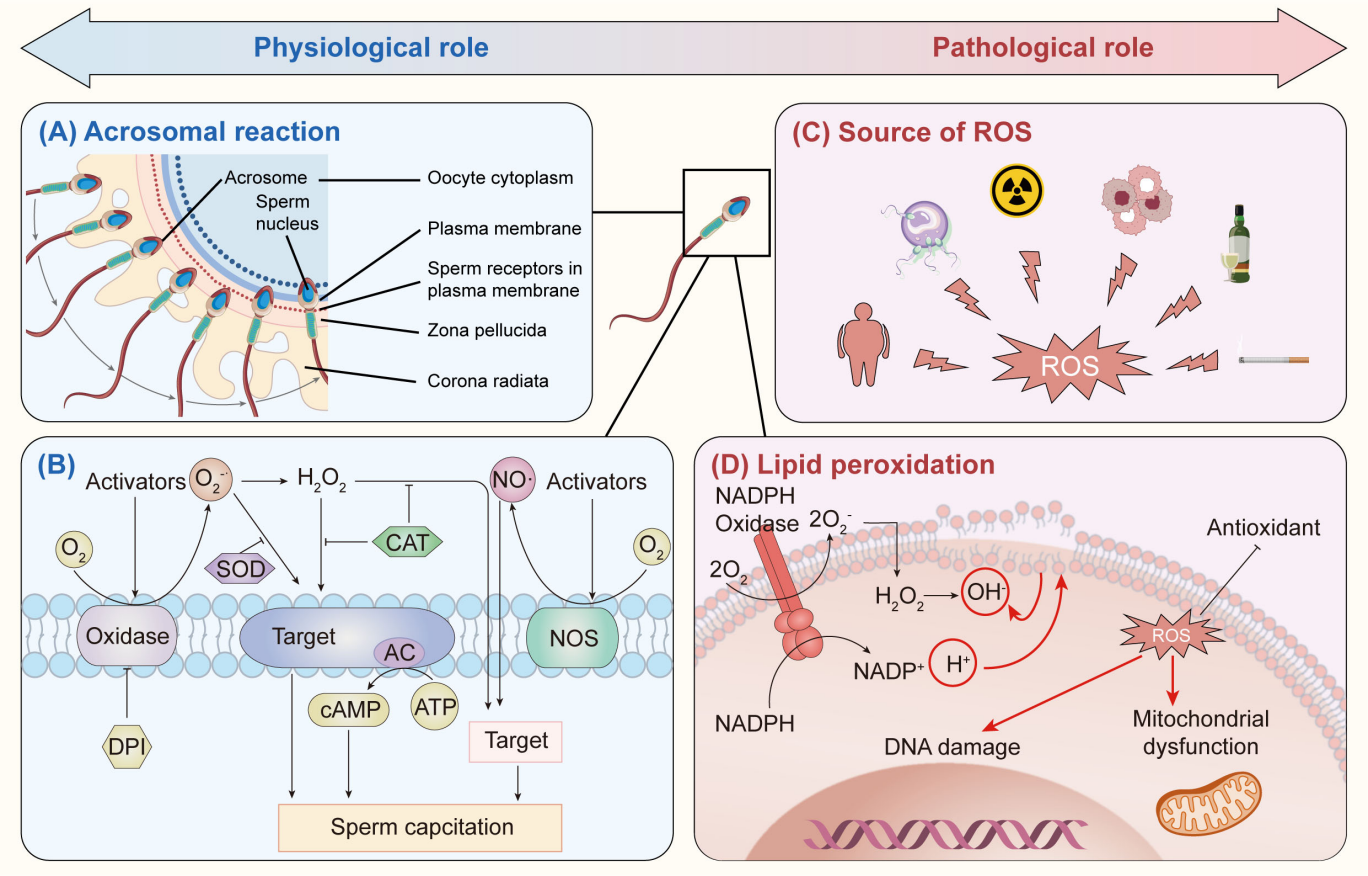
Reactive oxygen species (ROS) play both beneficial and harmful roles in the male reproductive system. **(A)** In small amounts, ROS are crucial for the acrosomal reaction and enable sperm to penetrate the zona pellucida through mild lipid peroxidation. **(B)** ROS are also involved in the early stages of sperm capacitation, with an oxidase producing extracellular superoxide (O_2_^•−^), which aids capacitation. Diphenyliodonium (DPI), an oxidase inhibitor, can block O_2_^•−^ production under oxidative stress, and SOD neutralizes excess O_2_^•−^. Additionally, hydrogen peroxide (H_2_O_2_) interacts with specific plasma membrane targets to support capacitation, while catalase prevents H_2_O_2_ overproduction. Nitric oxide (NO), generated by nitric oxide synthase (NOS), also promotes capacitation, with adenylyl cyclase (AC) as a possible ROS target, producing cAMP. **(C)** ROS sources in male infertility include obesity, immature sperm, radiation, leukocytosis, alcohol, and smoking. **(D)** NADPH oxidase in sperm membranes generates O_2_^•−^, which is converted to H_2_O_2_ in the cytosol, producing hydroxyl radicals (OH•). These radicals trigger lipid peroxidation, causing mitochondrial dysfunction and DNA damage in sperm.

Oxidative stress has become a central theme in understanding male infertility, particularly its connection with sperm dysfunction. The underlying biological phenomenon involves ROS, which play a complex dual role in sperm physiology and pathology. In controlled amounts, ROS are essential for various sperm functions, such as motility, capacitation, and fertilization (**Figure 1**). However, when the balance between ROS production and the body’s antioxidant defense systems is disrupted, ROS levels rise, resulting in oxidative stress (40, 41). This imbalance leads to structural and functional damage to spermatozoa, significantly reducing male fertility. Understanding how ROS affect sperm function and how oxidative stress leads to male infertility provides critical insights into potential therapeutic approaches. In healthy sperm, ROS serve vital physiological roles that are crucial for reproductive success. One of the primary functions of ROS is their involvement in sperm capacitation, a biochemical process that enables sperm to fertilize an egg. Capacitation is essential for the sperm to undergo hyperactivation, which is a type of vigorous motility required to navigate through the female reproductive tract and ultimately fertilize the oocyte. During capacitation, ROS facilitate the activation of key signal transduction pathways that regulate tyrosine phosphorylation, an essential process for sperm maturation. These pathways lead to alterations in the sperm membrane that increase its fluidity, thereby preparing the sperm for the acrosome reaction and fertilization. Another important function of ROS during capacitation is their role in cholesterol efflux from the sperm membrane (42). Cholesterol removal is necessary to increase membrane permeability and prepare the sperm for the acrosome reaction, which allows it to penetrate the egg’s outer layer (43, 44).

ROS are also involved in sperm motility, which is a critical determinant of male fertility. Motility is an energy-intensive process that requires the coordinated activity of several signaling pathways, particularly those involving calcium ion fluxes that regulate flagellar beating. ROS are known to modulate these pathways, ensuring that the sperm maintain their motility long enough to reach the oocyte. This balance between ROS levels and sperm motility is essential for successful fertilization. Physiological levels of ROS are also required for the acrosome reaction, which is the process by which sperm release enzymes that allow them to penetrate the zona pellucida, the protective layer surrounding the oocyte (45, 46). Without ROS, the acrosome reaction would not be triggered, and the sperm would be unable to fertilize the egg. The benefits of ROS in sperm function are clear under physiological conditions. However, their highly reactive nature means that even slight increases in ROS levels can have detrimental effects on sperm cells. This is where the importance of a tightly regulated balance between ROS production and antioxidant defenses becomes evident. Spermatozoa are highly specialized cells that are particularly vulnerable to oxidative damage due to their unique structural and functional characteristics. One of these characteristics is the high content of polyunsaturated fatty acids (PUFAs) in the sperm membrane (47, 48). PUFAs are particularly susceptible to oxidative attack by ROS, which can initiate a chain reaction of lipid peroxidation. This process not only damages the membrane’s structural integrity but also impairs its functionality, reducing the sperm’s ability to maintain motility and fertilize the egg.

The body’s antioxidant defense systems play a crucial role in preventing the harmful effects of excessive ROS. These systems include enzymatic antioxidants such as superoxide dismutase (SOD), catalase, and glutathione peroxidase, as well as non-enzymatic antioxidants like vitamins C and E, glutathione, and albumin (49, 50). Enzymatic antioxidants neutralize ROS by converting them into less reactive species. For instance, SOD converts superoxide anions into hydrogen peroxide, which is then broken down into water and oxygen by catalase and glutathione peroxidase (51, 52). Non-enzymatic antioxidants, on the other hand, act as scavengers that neutralize free radicals before they can cause cellular damage. In the male reproductive system, seminal plasma, the fluid that surrounds sperm, is rich in non-enzymatic antioxidants that protect sperm from oxidative damage. These antioxidants work in tandem with intracellular enzymatic defenses to maintain the fine balance between ROS and antioxidants, ensuring that ROS levels remain within a range that supports normal sperm function without causing oxidative stress.

When this balance is disrupted, however, oxidative stress occurs, leading to significant sperm dysfunction. The pathological effects of oxidative stress on spermatozoa can be grouped into three main categories: lipid peroxidation, DNA fragmentation, and protein oxidation. Each of these mechanisms contributes to reduced sperm quality and male infertility. Lipid peroxidation is one of the most damaging consequences of oxidative stress on sperm. As mentioned earlier, sperm membranes are rich in PUFAs, which are particularly prone to oxidation by ROS. When ROS levels rise, they initiate a chain reaction that leads to the formation of lipid peroxides. These peroxides, in turn, generate toxic byproducts such as MDA and 4-HNE, which further damage the sperm membrane (53–55). Lipid peroxidation reduces membrane fluidity, making it more difficult for sperm to swim effectively. It also impairs the integrity of the acrosomal membrane, which is necessary for the acrosome reaction and subsequent fertilization. Thus, lipid peroxidation has a direct impact on both sperm motility and the ability to fertilize the oocyte.

Another significant consequence of oxidative stress is DNA fragmentation. ROS can cause breaks in the DNA strands within sperm, leading to single-strand and double-strand breaks. These breaks often occur in regions of the sperm genome where chromatin is loosely packed, making them more susceptible to oxidative attack. DNA fragmentation is a major cause of male infertility, as it compromises the integrity of the paternal genome, leading to poor fertilization outcomes and an increased risk of genetic abnormalities in the offspring. Unlike somatic cells, spermatozoa have limited DNA repair mechanisms due to the replacement of histones with protamines during spermatogenesis. This limits their ability to repair oxidative damage, making DNA fragmentation particularly harmful. The use of assisted reproductive technologies (ART) such as *in vitro* fertilization (IVF) and intracytoplasmic sperm injection (ICSI) can bypass some of the natural selection mechanisms that would prevent sperm with damaged DNA from fertilizing an egg, potentially leading to long-term consequences for the offspring.

Protein oxidation is another detrimental effect of elevated ROS levels. ROS can modify key proteins involved in sperm motility, such as actin and tubulin, through the formation of carbonyl groups. These oxidative modifications can lead to the aggregation and dysfunction of these proteins, compromising the structural integrity of the sperm’s cytoskeleton and impairing motility. Protein oxidation can also affect enzymes that regulate sperm metabolism, reducing the availability of ATP, which is required for motility and fertilization. Moreover, the oxidation of surface proteins on the sperm membrane can impair the sperm’s ability to recognize and bind to the oocyte, further reducing the likelihood of successful fertilization. The sources of oxidative stress in sperm can be both intrinsic and extrinsic. Intrinsic sources include the mitochondria, which are a major source of ROS during the process of oxidative phosphorylation. Mitochondria are responsible for producing the energy required for sperm motility, but during this process, some electrons leak from the electron transport chain and react with oxygen to form superoxide anions (56, 57). This mitochondrial ROS production is a natural part of cellular metabolism, but when ROS levels are excessive, they can damage the mitochondria themselves, leading to a reduction in ATP production and further impairing sperm motility. Moreover, mitochondrial DNA, which lacks the protective histones found in nuclear DNA, is particularly vulnerable to oxidative damage. Mutations in mitochondrial DNA caused by ROS can exacerbate mitochondrial dysfunction, creating a vicious cycle of oxidative stress and impaired sperm function.

Extrinsic sources of oxidative stress include environmental and lifestyle factors. Exposure to environmental toxins such as heavy metals (e.g., cadmium, lead) and endocrine-disrupting chemicals (e.g., bisphenol A, phthalates) can increase ROS production in spermatozoa. Lifestyle factors such as smoking, excessive alcohol consumption, poor diet, and obesity are also major contributors to oxidative stress (58, 59). Smoking, for instance, introduces numerous free radicals into the body, which increase ROS levels and cause oxidative damage to sperm DNA, proteins, and lipids (60, 61). Infections and inflammation in the male reproductive tract can also lead to elevated ROS levels due to the activation of leukocytes, which produce ROS as part of the body’s immune response. This condition, known as leukocytospermia, has been associated with increased oxidative stress and sperm DNA fragmentation.

In conclusion, ROS play a dual role in sperm function, being essential for processes such as capacitation, motility, and fertilization at physiological levels, but harmful when produced in excess. The balance between ROS production and antioxidant defenses is crucial for maintaining sperm health and function. When this balance is disrupted, oxidative stress occurs, leading to lipid peroxidation, DNA fragmentation, and protein oxidation, all of which contribute to sperm dysfunction and male infertility. The pathological effects of oxidative stress underscore the importance of developing therapeutic strategies aimed at reducing ROS levels and enhancing antioxidant defenses to improve male fertility outcomes.

## 3 Molecular mechanisms of oxidative stress-induced sperm damage

Oxidative stress has been extensively recognized as a significant contributor to male infertility, particularly through its damaging effects on spermatozoa. As one of the most vulnerable cell types in the human body, spermatozoa possess unique structural and functional characteristics that make them highly susceptible to oxidative damage. This vulnerability is primarily due to the high content of PUFAs in their membranes, the limited availability of antioxidant enzymes, and the lack of effective DNA repair mechanisms. When ROS levels exceed the antioxidant defenses, spermatozoa are subjected to oxidative stress, leading to detrimental molecular changes, including lipid peroxidation, DNA fragmentation, and protein oxidation. These molecular alterations compromise sperm function, reducing their motility, fertilization capacity, and genetic integrity. This section delves into the specific molecular mechanisms by which oxidative stress induces sperm damage, focusing on lipid peroxidation, DNA fragmentation, and protein oxidation.

### 3.1 Lipid peroxidation

One of the earliest and most significant effects of oxidative stress on spermatozoa is lipid peroxidation (62, 63). This process involves the oxidative degradation of lipids, specifically PUFAs present in the sperm membrane. PUFAs, with their multiple double bonds, are highly susceptible to attack by ROS, making spermatozoa particularly vulnerable to oxidative stress. Lipid peroxidation results in the formation of lipid peroxides and various toxic byproducts, which lead to a cascade of cellular damage, ultimately impairing sperm function. Lipid peroxidation occurs when ROS, particularly hydroxyl radicals (•OH) and superoxide anions (O_2_^−^), interact with the PUFAs in sperm membranes. The process is initiated when ROS abstract a hydrogen atom from the methylene group of a PUFA, creating a lipid radical. This lipid radical reacts with molecular oxygen to form lipid peroxyl radicals, which perpetuate a chain reaction of oxidative damage. As lipid peroxyl radicals propagate the reaction, they generate lipid hydroperoxides and highly toxic byproducts such as MDA and 4-HNE (54, 64). These byproducts further damage membrane lipids, proteins, and even DNA, amplifying the effects of oxidative stress.

The impact of lipid peroxidation on sperm function is profound. The sperm membrane plays a crucial role in maintaining the structural integrity and functionality of the cell. The lipid bilayer, which is rich in PUFAs, ensures membrane fluidity, which is essential for sperm motility and the ability to undergo capacitation and the acrosome reaction—two key processes required for successful fertilization. Lipid peroxidation compromises membrane fluidity, leading to a reduction in sperm motility. This occurs because the lipid peroxides generated during the process disrupt the normal lipid packing in the membrane, making it more rigid and less flexible. As a result, the sperm’s ability to propel itself through the female reproductive tract is impaired, significantly reducing the chances of reaching and fertilizing the oocyte. Furthermore, lipid peroxidation damages the sperm’s ability to fuse with the oocyte. The acrosomal membrane, located at the head of the sperm, is responsible for releasing hydrolytic enzymes during the acrosome reaction, which allows the sperm to penetrate the zona pellucida, the outer layer of the egg. When lipid peroxidation affects the acrosomal membrane, the acrosome reaction is disrupted, preventing the sperm from binding to and fertilizing the egg. Clinically, lipid peroxidation serves as a biomarker of oxidative stress in spermatozoa. Elevated levels of MDA and 4-HNE in semen samples are often correlated with reduced sperm quality and increased infertility rates. These markers are used in diagnostic assessments to evaluate the extent of oxidative damage and to guide antioxidant therapy aimed at reducing lipid peroxidation and improving sperm function.

### 3.2 DNA fragmentation

**Figure 2:**
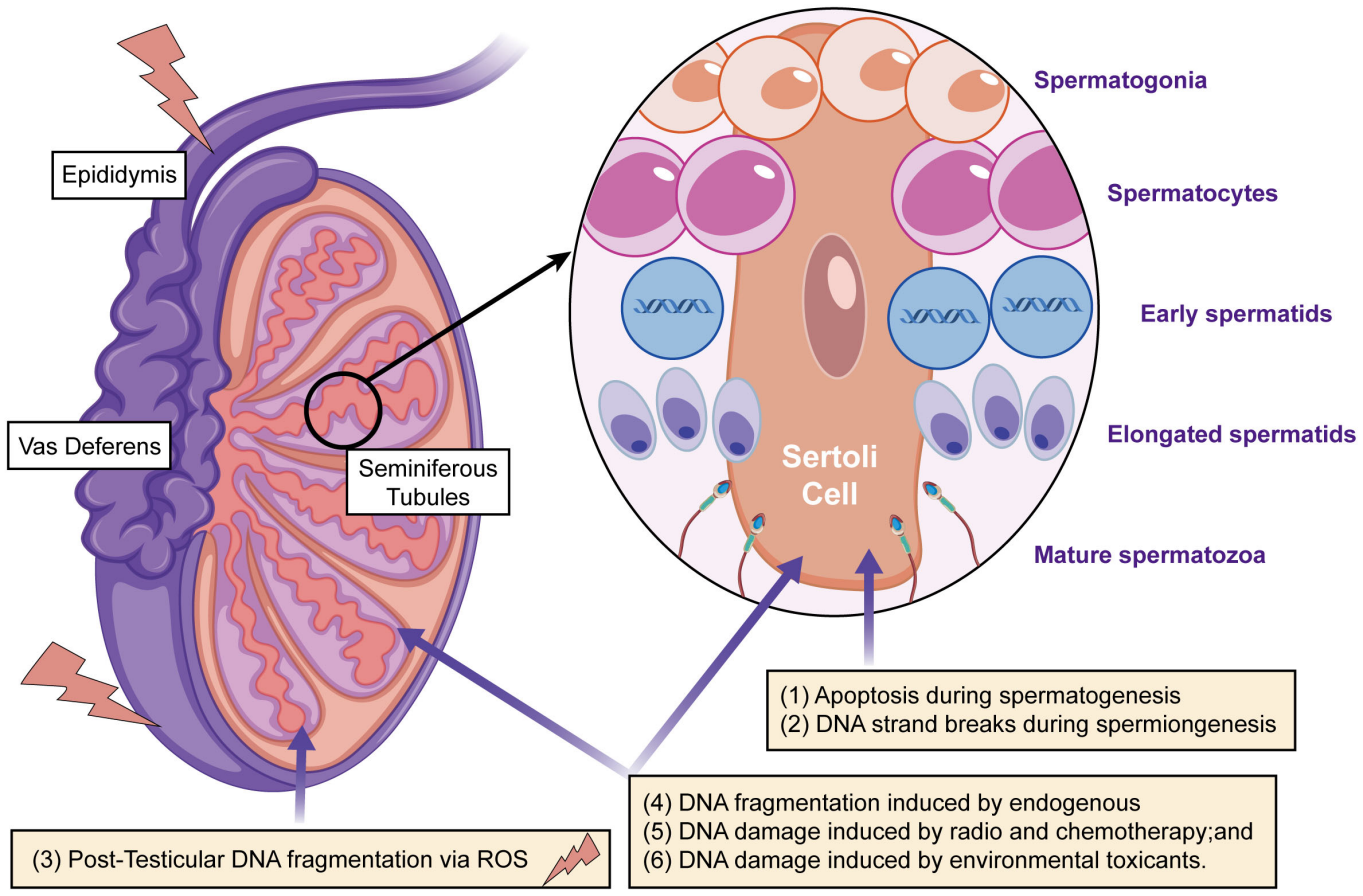
The main mechanisms that cause DNA damage in sperm during production or transport include: (i) apoptosis occurring during spermatogenesis; (ii) DNA strand breaks during chromatin remodeling in spermiogenesis; (iii) post-testicular DNA fragmentation, primarily due to oxidative stress, during sperm transit through the seminiferous tubules and epididymis; (iv) DNA fragmentation caused by internal enzymes like caspases and endonucleases; (v) DNA damage from radiotherapy and chemotherapy; and (vi) DNA damage resulting from exposure to environmental toxins.

In addition to lipid peroxidation, oxidative stress induces significant damage to sperm DNA, leading to DNA fragmentation (**Figure 2**). Spermatozoa are unique in that their DNA is highly compacted and tightly packaged within protamines, which replace histones during spermatogenesis. This compaction provides a degree of protection against external damage; however, sperm DNA remains vulnerable to oxidative stress due to the limited availability of DNA repair mechanisms and the loose packaging of DNA at certain sites, particularly those involved in early embryonic development. Oxidative damage to sperm DNA occurs through the formation of single-strand and double-strand breaks, as well as the generation of oxidative base adducts. ROS, particularly hydroxyl radicals, interact with DNA bases, leading to base modifications, strand breaks, and the formation of oxidative lesions. One of the most well-documented oxidative DNA adducts is 8-OHdG, which results from the oxidation of guanine (65, 66). 8-OHdG serves as a biomarker of oxidative stress in spermatozoa and has been linked to chromosomal instability and mutagenesis (67, 68).

The process of DNA fragmentation begins when ROS attack the deoxyribose backbone of DNA, leading to the cleavage of phosphodiester bonds and the formation of strand breaks (69, 70). These breaks can occur as single-strand or double-strand breaks, both of which compromise the integrity of the sperm genome. Single-strand breaks may be repaired by the oocyte after fertilization, but double-strand breaks are more difficult to repair and are often associated with severe chromosomal damage. DNA fragmentation has a direct impact on sperm function and male fertility. Spermatozoa with fragmented DNA exhibit reduced fertilization capacity because the damaged DNA impairs the sperm’s ability to effectively deliver the paternal genome to the oocyte (71, 72). Even if fertilization occurs, the presence of fragmented DNA increases the likelihood of poor reproductive outcomes, including failed implantation, miscarriage, and the development of genetic abnormalities in the offspring. Studies have shown that sperm DNA fragmentation is strongly correlated with recurrent pregnancy loss, particularly in couples undergoing ART such as IVF and ICSI.

One of the major challenges in addressing oxidative DNA damage in sperm is the limited DNA repair capacity of spermatozoa. Unlike somatic cells, which possess a variety of DNA repair pathways to correct oxidative damage, spermatozoa have only one active DNA repair enzyme, 8-oxoguanine DNA glycosylase (OGG1), which is responsible for repairing oxidative base lesions (73, 74). The limited repair capacity of sperm makes them highly susceptible to accumulating DNA damage, particularly in the context of oxidative stress. While the oocyte possesses more robust DNA repair mechanisms, its ability to repair sperm DNA damage is finite and decreases with maternal age. Therefore, sperm DNA fragmentation represents a critical barrier to successful reproduction, particularly in older couples or those experiencing male factor infertility. The clinical implications of sperm DNA fragmentation are significant. Sperm DNA fragmentation testing is commonly used to assess male fertility potential, particularly in cases of unexplained infertility, recurrent pregnancy loss, and poor ART outcomes. Therapeutic strategies to reduce sperm DNA fragmentation typically focus on antioxidant supplementation, which aims to neutralize ROS and reduce oxidative damage to DNA. However, given the limited DNA repair capacity of sperm, early intervention is crucial to prevent irreversible damage.

### 3.3 Protein oxidation

Protein oxidation represents another key mechanism by which oxidative stress impairs sperm function. ROS can modify sperm proteins through the oxidation of amino acid side chains, leading to the formation of carbonyl groups and disulfide bonds. These oxidative modifications result in the aggregation and dysfunction of key proteins involved in sperm motility, structure, and fertilization, further contributing to male infertility. The process of protein oxidation begins when ROS interact with specific amino acids, such as cysteine, methionine, and tyrosine, which contain sulfur or aromatic groups that are highly susceptible to oxidation. This interaction leads to the formation of protein carbonyls and disulfide bonds, which alter the protein’s structure and function. In spermatozoa, the proteins most affected by oxidative stress are those involved in maintaining the cytoskeleton, motility, and the acrosome reaction.

One of the primary targets of protein oxidation in sperm is the cytoskeletal proteins actin and tubulin (75). These proteins form the structural framework of the sperm flagellum, which is responsible for motility. Oxidation of actin and tubulin disrupts the integrity of the cytoskeleton, leading to impaired flagellar movement and reduced sperm motility. Sperm motility is essential for navigating the female reproductive tract and reaching the oocyte for fertilization. When motility is compromised due to protein oxidation, the sperm’s ability to reach and fertilize the egg is significantly diminished. In addition to cytoskeletal proteins, enzymes involved in sperm metabolism and energy production are also affected by oxidative stress (75, 76). These enzymes, such as creatine kinase and adenylate kinase, play critical roles in generating the ATP required for sperm motility and the acrosome reaction (77, 78). Oxidative damage to these enzymes reduces their activity, leading to decreased ATP production and impaired sperm function. Without sufficient energy, spermatozoa cannot sustain the vigorous motility required for successful fertilization.

Protein oxidation also affects the acrosome reaction, which is essential for sperm-egg binding and fertilization. The acrosome is a cap-like structure located at the head of the sperm that contains hydrolytic enzymes necessary for penetrating the zona pellucida. Oxidative damage to proteins involved in the acrosome reaction, such as acrosin and hyaluronidase, impairs the sperm’s ability to release these enzymes, preventing successful penetration of the oocyte (79, 80). As a result, the sperm’s fertilization capacity is significantly reduced. Proteomics approaches have been employed to detect oxidative modifications in sperm proteins. These techniques involve the identification and quantification of protein carbonyls and other oxidative markers, providing valuable insights into the extent of oxidative damage in spermatozoa. By identifying specific proteins that are oxidized in response to oxidative stress, researchers can better understand the molecular mechanisms underlying sperm dysfunction and male infertility. The clinical implications of protein oxidation in male infertility are significant. Oxidative damage to sperm proteins not only impairs motility and fertilization but also contributes to the overall decline in sperm quality observed in infertile men. Therapeutic interventions aimed at reducing protein oxidation typically focus on antioxidant supplementation, which has been shown to improve sperm motility and reduce oxidative damage in clinical studies.

## 4 Sources of oxidative stress in sperm

Oxidative stress in spermatozoa occurs when there is a disproportion between the generation of ROS and the capacity of the available antioxidant defenses to neutralize them. While controlled ROS levels are essential for normal sperm processes—such as capacitation, motility, and the fertilization mechanism—excessive ROS accumulation leads to oxidative damage, impairing sperm function and ultimately contributing to male infertility. The origins of oxidative stress in sperm are both internal and external, with various physiological, pathological, and environmental influences promoting elevated ROS production. In this section, we will examine the key contributors to oxidative stress in sperm, concentrating on ROS produced by leukocytes, mitochondrial dysfunction, and environmental factors.

### 4.1 Leukocyte-derived ROS

Leukocytes, or white blood cells, are an important component of the immune system and are often present in semen, particularly in response to infections or inflammatory conditions in the male reproductive tract (81, 82). While leukocytes are essential for immune defense, their activation in the reproductive environment can lead to excessive production of ROS, which in turn can damage spermatozoa. This oxidative stress from leukocyte-derived ROS is a well-established factor in male infertility, especially in cases associated with leukocytospermia, a condition characterized by elevated leukocyte counts in semen. When infections or inflammatory responses occur within the male reproductive system, leukocytes—particularly neutrophils and macrophages—become activated and initiate a process known as the respiratory burst (83, 84). During this process, activated leukocytes produce large amounts of ROS as part of their mechanism to destroy invading pathogens. Hypochlorous acid is a potent oxidant used by leukocytes to kill bacteria, but in the context of the reproductive environment, it can have deleterious effects on sperm cells.

The ROS generated by leukocytes are not restricted to attacking pathogens; they can also interact with nearby spermatozoa, leading to oxidative damage. ROS from leukocytes can induce lipid peroxidation in sperm membranes, leading to decreased membrane fluidity, impaired motility, and compromised sperm-oocyte fusion. Additionally, ROS can cause DNA fragmentation in sperm, reducing genetic integrity and fertilization potential. Leukocytospermia, characterized by the presence of more than one million leukocytes per milliliter of semen, is a well-established contributor to male infertility. In such cases, the excessive production of ROS by leukocytes surpasses the capacity of the semen’s antioxidant defenses, leading to pronounced oxidative stress. Research has demonstrated that men with leukocytospermia exhibit higher levels of sperm DNA fragmentation, diminished sperm motility, and reduced fertilization rates, all of which are significant factors contributing to infertility (85, 86). This imbalance between ROS production and antioxidant capacity in the presence of leukocytes underscores the critical role of managing oxidative stress in treating male infertility (87, 88).

To mitigate leukocyte-induced oxidative stress in sperm, several strategies have been proposed. Anti-inflammatory treatments, such as the use of nonsteroidal anti-inflammatory drugs (NSAIDs) or corticosteroids, can help reduce leukocyte activation and ROS production. In cases where infections are the underlying cause of leukocytospermia, appropriate antimicrobial therapy can reduce leukocyte infiltration and ROS generation. Antioxidant supplementation is another approach used to counteract the effects of leukocyte-derived ROS. Vitamins C and E, glutathione, and coenzyme Q10 are examples of antioxidants that can neutralize ROS and reduce oxidative damage to sperm. Additionally, lifestyle modifications—such as reducing exposure to environmental toxins and managing stress—can help lower inflammation and reduce leukocyte activation.

### 4.2 Mitochondrial dysfunction

Mitochondria are another important internal source of ROS in sperm cells. As the cell’s energy producers, mitochondria create ATP through a process called oxidative phosphorylation, which uses the electron transport chain (ETC) (41, 89). However, during this process, some electrons can escape from the ETC and react with oxygen, forming superoxide anions (O_2_^−^), a type of ROS. Although mitochondria have antioxidant defenses like SOD, which helps convert superoxide anions into less harmful molecules, too much mitochondrial ROS can cause oxidative stress and harm both the mitochondria and the sperm cell. Mitochondrial problems in sperm are a significant factor in male infertility, as they interfere with energy production and the balance needed for sperm function. Located in the midpiece of the sperm, mitochondria are essential for providing the energy that enables sperm movement. When ROS levels in mitochondria are elevated due to electron leakage from the ETC, mitochondrial superoxide is generated, initiating a cascade of oxidative damage (35, 90). This oxidative stress can impair mitochondrial function, leading to reduced ATP production, which is necessary for sperm motility. As a result, sperm with dysfunctional mitochondria exhibit decreased motility and an inability to reach the oocyte, significantly reducing fertility potential.

In addition to impaired energy production, mitochondrial dysfunction can lead to direct damage to mitochondrial DNA (mtDNA) (91, 92). Furthermore, the limited DNA repair capacity in mitochondria makes it difficult for mtDNA to recover from oxidative damage. Mutations in mtDNA caused by ROS can further exacerbate mitochondrial dysfunction, creating a vicious cycle of oxidative stress and energy deficits (40, 41). This mitochondrial damage has been strongly associated with asthenozoospermia, a condition characterized by reduced sperm motility, which is a common cause of male infertility. The link between mitochondrial dysfunction and male infertility has been well-established in both clinical and experimental studies. Men with infertility often exhibit higher levels of mitochondrial ROS and lower levels of mitochondrial membrane potential, which correlates with poor sperm motility and reduced fertilization rates. Addressing mitochondrial dysfunction and reducing mitochondrial ROS production are important therapeutic goals for improving sperm quality and male fertility. Antioxidant therapies that specifically target mitochondria, such as coenzyme Q10 and mitochondrial-targeted antioxidants (e.g., mitoquinone), have shown promise in reducing mitochondrial oxidative stress and improving sperm motility (93). These antioxidants help restore the redox balance within mitochondria, reduce oxidative damage to mtDNA, and enhance ATP production, ultimately improving sperm function and fertility outcomes.

### 4.3 External and environmental sources

In addition to endogenous sources of oxidative stress, spermatozoa are also exposed to a variety of external and environmental factors that contribute to elevated ROS levels. These factors include pollution, exposure to toxins, lifestyle choices, and dietary habits, all of which have been shown to significantly impact sperm quality and fertility. Environmental pollution is a major contributor to oxidative stress in sperm. Airborne pollutants like particulate matter (PM), polycyclic aromatic hydrocarbons (PAHs), and heavy metals (such as cadmium and lead) can trigger ROS production when they are inhaled or absorbed into the body. These pollutants can harm sperm cells directly or cause systemic inflammation and oxidative stress in the male reproductive system. For example, exposure to PAHs and heavy metals has been linked to higher ROS levels, lower sperm motility, and increased DNA fragmentation in sperm. Similarly, men who are exposed to chemicals, pesticides, or solvents at work have been found to experience greater oxidative damage in their sperm, leading to reduced fertility.

Lifestyle factors, particularly smoking and excessive alcohol consumption, are also significant contributors to oxidative stress in spermatozoa. Cigarette smoke contains a variety of toxic substances, including free radicals and heavy metals, that generate ROS both directly in the male reproductive tract and indirectly through systemic oxidative stress. Studies have consistently shown that smokers have higher levels of sperm DNA fragmentation, reduced sperm motility, and lower antioxidant capacity compared to non-smokers. Alcohol consumption exacerbates oxidative stress by depleting antioxidant reserves, such as glutathione, and increasing the production of ROS through the metabolism of ethanol. Diet and nutrition also play a key role in modulating oxidative stress in sperm. Diets high in saturated fats and processed foods can lead to increased inflammation and oxidative stress, while diets rich in antioxidants like vitamins C and E, zinc, selenium, and omega-3 fatty acids help protect sperm from oxidative damage. Studies show that consuming antioxidant-rich foods and supplements can improve sperm motility, reduce DNA fragmentation, and boost overall sperm quality in men with infertility. On the other hand, unhealthy eating habits that contribute to obesity and metabolic syndrome are linked to higher oxidative stress, more inflammation, and decreased sperm function (94).

Another significant source of external oxidative stress is exposure to heat and radiation. Elevated scrotal temperature, caused by factors such as prolonged sitting, tight clothing, or the use of electronic devices, can increase ROS production in spermatozoa and reduce sperm motility. Similarly, exposure to ionizing radiation, such as from medical imaging or environmental sources, can generate ROS and induce oxidative DNA damage in sperm, leading to reduced fertility. Mitigating the effects of external and environmental sources of oxidative stress on sperm requires a multifaceted approach. Reducing exposure to environmental pollutants and toxins, adopting healthy lifestyle habits, and improving dietary choices can significantly reduce ROS levels and improve sperm quality.

Oxidative stress in spermatozoa arises from a combination of endogenous and exogenous sources, with leukocyte-derived ROS, mitochondrial dysfunction, and external environmental factors all playing significant roles in generating ROS and damaging sperm cells. Leukocyte-derived ROS, particularly in the context of infections and inflammation, can induce lipid peroxidation, DNA fragmentation, and protein oxidation, all of which contribute to reduced sperm quality and male infertility. Mitochondrial dysfunction further exacerbates oxidative stress by impairing ATP production and damaging mitochondrial DNA, leading to compromised sperm motility and energy deficits. External factors, including pollution, toxins, lifestyle choices, and dietary habits, also contribute to oxidative stress and can significantly impact sperm quality and fertility outcomes. Understanding these sources of oxidative stress is essential for developing targeted therapeutic interventions, including antioxidant supplementation, anti-inflammatory treatments, and lifestyle modifications, to improve sperm function and enhance male fertility (95).

## 5 Antioxidant defense mechanisms in sperm

Spermatozoa are uniquely specialized cells essential for the process of fertilization, yet they exhibit a pronounced susceptibility to oxidative stress due to their distinct biological properties. Their membranes are rich in polyunsaturated fatty acids (PUFAs), which are crucial for preserving membrane fluidity and functionality. However, these PUFAs are particularly prone to oxidative damage from ROS. Compounding this vulnerability, sperm cells contain minimal cytoplasm, thereby limiting their capacity to store antioxidant enzymes and molecules that could neutralize ROS. Consequently, spermatozoa rely extensively on both enzymatic and non-enzymatic antioxidant defense systems to mitigate oxidative damage. This section will delve into the available antioxidant defenses in sperm cells, their inherent limitations, and the therapeutic approaches designed to bolster these protective mechanisms.

### 5.1 Natural antioxidant defenses in sperm

The body inherently generates antioxidant enzymes and non-enzymatic molecules to neutralize ROS and shield cells from oxidative damage. In spermatozoa, several key antioxidant enzymes are present, including SOD, glutathione peroxidase (GPx), and catalase. These enzymes are essential for scavenging ROS and mitigating the harmful effects of oxidative stress. Superoxide dismutase catalyzes the conversion of superoxide radicals into hydrogen peroxide, which is then broken down by catalase and GPx into water and oxygen, thereby preventing the accumulation of toxic byproducts and preserving the structural and functional integrity of sperm cells. These antioxidant defenses are vital for protecting spermatozoa from the detrimental consequences of oxidative stress, which can impair motility, fertilization potential, and DNA integrity (52, 96). These antioxidant enzymes work in concert to detoxify ROS, thereby reducing their harmful impact on sperm cells. Superoxide dismutase is one of the most important enzymatic antioxidants present in sperm. It catalyzes the conversion of superoxide anions (O_2_^−^), which are highly reactive, into hydrogen peroxide (H_2_O_2_), which is less harmful. This process is crucial because superoxide anions are among the first ROS to be produced during oxidative stress, particularly in mitochondria, which are key sources of ROS in sperm cells. By converting superoxide anions into hydrogen peroxide, SOD prevents them from reacting with other molecules and causing further damage (97, 98).

Glutathione peroxidase (GPx) serves as another essential antioxidant enzyme in sperm, playing a pivotal role in neutralizing hydrogen peroxide and lipid hydroperoxides, which arise when ROS target polyunsaturated fatty acids (PUFAs) in the sperm membrane (99, 100). GPx relies on glutathione (GSH), a tripeptide that donates electrons to reduce peroxides and counteract ROS, effectively preventing oxidative damage. By inhibiting lipid peroxidation, GPx safeguards the structural integrity of the sperm membrane, thereby maintaining motility and preserving the sperm’s fertilization potential. Catalase also plays a crucial part in the sperm’s antioxidant defense system. Similar to GPx, catalase converts hydrogen peroxide into water and oxygen, thus preventing the buildup of hydrogen peroxide and inhibiting the formation of highly reactive hydroxyl radicals (•OH) via the Fenton reaction (101, 102). Hydroxyl radicals, among the most destructive ROS, can inflict severe oxidative damage on DNA, proteins, and lipids within sperm cells. By detoxifying hydrogen peroxide, catalase helps maintain sperm functionality and protects against oxidative stress-induced damage, further reinforcing the cell’s defense mechanisms.

In addition to these enzymatic antioxidants, sperm and seminal plasma contain several non-enzymatic antioxidants that provide further protection against oxidative stress. These include vitamins C and E, glutathione, coenzyme Q10, selenium, and zinc. Vitamin C (ascorbic acid) is a water-soluble antioxidant that acts as a primary line of defense against ROS in the seminal plasma. It scavenges free radicals and helps regenerate vitamin E, which is a lipid-soluble antioxidant that protects the sperm membrane from lipid peroxidation. Vitamin E, in particular, is essential for maintaining the integrity of the PUFAs in the sperm membrane, which are highly susceptible to oxidative attack. Glutathione, which works in tandem with GPx, plays a critical role in reducing lipid hydroperoxides and protecting sperm DNA from oxidative damage. Selenium and zinc support the activity of key antioxidant enzymes, including GPx and SOD, helping to enhance sperm antioxidant defenses. Coenzyme Q10, which is involved in mitochondrial energy production, also functions as an antioxidant by reducing mitochondrial ROS production and protecting sperm from oxidative stress. These antioxidants play a vital role in mitigating the oxidative stress induced by the freezing-thawing process, improving sperm viability post-thaw, and reducing DNA fragmentation. For instance, adding antioxidants to cryopreservative media has been shown to enhance sperm motility and fertilizing capacity after thawing, contributing to higher pregnancy rates in ART. Additionally, antioxidants can protect sperm DNA integrity, reducing fragmentation caused by ROS during sperm processing, which is crucial for successful fertilization and embryo development.

### 5.2 Limitations of sperm’s antioxidant capacity

Antioxidants play a critical role in protecting cells from oxidative damage, which can severely affect key cellular components like the plasma membrane, proteins, and DNA. Various techniques are employed to detect ROS, each offering unique insights into their impact on cellular structures. For example, fluorescent probes such as DCFDA and DHE are commonly used to visualize ROS generation in live cells. These probes emit fluorescence upon oxidation, allowing real-time monitoring of ROS levels in various cellular compartments. Another powerful technique is electron spin resonance (ESR), which directly detects free radicals and is often used in combination with spin-trapping agents to enhance the sensitivity of ROS detection. Chemiluminescence assays can also quantify ROS levels in biological samples, providing a sensitive method for assessing oxidative stress. Moreover, HPLC-MS/MS techniques allow for the precise identification and quantification of ROS byproducts, providing detailed information on the types of oxidative damage occurring within the cell. The damage caused by ROS extends to the plasma membrane, where lipid peroxidation is a primary consequence. Lipid peroxidation can be measured by the TBARS assay or through the detection of MDA, a byproduct of membrane lipid oxidation. This damage compromises membrane integrity, affecting cellular signaling and transport functions. At the protein level, ROS can induce oxidation of amino acids, particularly cysteine and methionine residues. Techniques such as Western blotting and mass spectrometry are often used to identify and quantify protein carbonylation, a hallmark of oxidative protein damage. Finally, DNA is highly susceptible to ROS-induced damage, which can result in strand breaks, base modifications, and crosslinking. The Comet assay is a widely used technique to assess DNA strand breaks at the single-cell level, providing valuable insight into the extent of genotoxic damage caused by ROS. Additionally, the use of immunofluorescence assays to detect γH2AX foci offers a powerful approach for identifying double-strand breaks in DNA, a direct consequence of ROS-mediated damage.

Despite having these natural antioxidant defenses, spermatozoa are highly susceptible to oxidative stress due to several inherent limitations in their ability to counteract excessive ROS. One of the primary limitations is the reduced cytoplasmic volume in sperm cells, which limits the storage of antioxidant enzymes and molecules. During spermatogenesis, sperm lose most of their cytoplasm to achieve a streamlined shape that facilitates motility. However, this reduction in cytoplasm also reduces the capacity of sperm to harbor antioxidant enzymes, making them more vulnerable to oxidative stress. Compared to somatic cells, sperm cells have fewer resources to neutralize ROS, leaving them more exposed to oxidative damage.

Another limitation is the high content of PUFAs in sperm membranes. While these fatty acids are essential for maintaining membrane fluidity and ensuring that sperm can navigate the female reproductive tract and fertilize the oocyte, they are also highly prone to oxidation. When ROS levels rise, PUFAs in the sperm membrane are oxidized in a process known as lipid peroxidation, which generates toxic byproducts such as MDA and 4-HNE. These byproducts can damage the sperm membrane, reducing its fluidity and impairing sperm motility and function. Once lipid peroxidation is initiated, it can propagate rapidly, causing widespread membrane damage that significantly impairs sperm’s ability to fertilize the egg. In addition to limited antioxidant reserves and the vulnerability of PUFAs, sperm mitochondria are another source of ROS that contributes to oxidative stress. Mitochondria are responsible for producing the energy (in the form of ATP) required for sperm motility through the process of oxidative phosphorylation. While mitochondria have their own antioxidant defenses, including mitochondrial SOD, these defenses are often insufficient to neutralize the high levels of ROS produced during oxidative phosphorylation, especially if mitochondrial function is compromised. The continuous production of ROS by mitochondria can lead to mitochondrial dysfunction, impaired ATP production, and reduced sperm motility.

Another major limitation of spermatozoa is their inability to effectively repair DNA damage. During spermatogenesis, sperm DNA is highly compacted and packaged with protamines, replacing histones to create a more condensed structure. While this packaging protects the DNA from some forms of damage, it also limits the sperm cell’s ability to repair oxidative damage, such as DNA fragmentation caused by ROS.

### 5.3 Therapeutic strategies to enhance antioxidant defense

Given the limitations of sperm’s natural antioxidant defenses, therapeutic strategies aimed at enhancing antioxidant capacity are essential for protecting sperm from oxidative stress and improving male fertility (103). Antioxidant supplementation is one of the most widely studied and applied approaches to enhancing sperm antioxidant defenses. A variety of antioxidant supplements, including vitamins C, glutathione, selenium, and zinc, have been shown to improve sperm quality by reducing ROS levels and preventing oxidative damage (104, 105). These antioxidants work by scavenging ROS, reducing lipid peroxidation, and protecting DNA and proteins from oxidative stress-induced damage. Antioxidants, including vitamin C, vitamin E, and Coenzyme Q10, play a critical role in protecting cells from oxidative stress (106, 107). In fertility treatments, antioxidants help to safeguard the DNA integrity of sperm and eggs, which is essential for successful conception. Research has shown that antioxidants can enhance egg quality in women undergoing IVF treatments and improve sperm motility in men, offering a practical solution for couples struggling with infertility (108, 109). For instance, CoQ10 supplementation has been linked to improved egg quality and better outcomes in IVF patients (110, 111). Furthermore, the use of antioxidants in animal conservation efforts, such as enhancing reproductive health in endangered species, is gaining traction. By mitigating oxidative damage in animals exposed to captivity stress or environmental toxins, antioxidants have been used to improve breeding success rates, exemplified by their use in endangered amphibian programs. These practical applications highlight the growing significance of antioxidants not only in human fertility treatments but also in global biodiversity conservation efforts.

In addition to supplementation, dietary and lifestyle modifications can also help enhance sperm antioxidant defenses. A diet rich in antioxidant-rich foods, such as fruits, vegetables, nuts, and seeds, provides a wide range of vitamins, minerals, and polyphenols that help neutralize ROS and protect sperm from oxidative damage. Lifestyle changes, such as quitting smoking, reducing alcohol intake, and avoiding environmental toxins, can also reduce ROS production and improve sperm quality. Smoking and alcohol consumption are known to increase oxidative stress, deplete antioxidant reserves, and impair sperm function. By making these lifestyle changes, men can significantly reduce their oxidative stress levels and improve their reproductive health.

In conclusion, while sperm possess natural antioxidant defense mechanisms, these defenses are limited by the unique characteristics of spermatozoa, including their reduced cytoplasmic volume, high content of PUFAs, and mitochondrial ROS production. Enhancing antioxidant defenses through supplementation, dietary modifications, and lifestyle changes is a promising approach to reducing oxidative stress, protecting sperm from damage, and improving male fertility outcomes. Understanding the limitations of sperm’s antioxidant capacity and implementing strategies to boost these defenses is essential for addressing oxidative stress-related male infertility.

## 6 Conclusion

Oxidative stress is broadly acknowledged as a principal contributor to male infertility, exerting a substantial influence on spermatozoal dysfunction. As emphasized in the discourse, this stress emerges from a disproportion between the generation of ROS and the organism’s capacity to counteract these reactive entities via its antioxidant mechanisms. Sperm cells, in particular, are highly susceptible to oxidative stress because of their membranes’ rich PUFA content, coupled with a scarcity of antioxidant protections and a restricted ability for DNA repair. The molecular mechanisms by which oxidative stress induces sperm damage are diverse and have profound implications for male fertility. These mechanisms include lipid peroxidation, DNA fragmentation, and protein oxidation, all of which lead to compromised sperm function, reduced motility, impaired fertilization capability, and genetic abnormalities that may affect offspring. Lipid peroxidation stands out as one of the most critical outcomes of oxidative stress affecting sperm function. This process entails the oxidation of polyunsaturated fatty acids (PUFAs) within the sperm membrane, giving rise to harmful byproducts such as MDA and 4-HNE. These toxic compounds compromise the fluidity and integrity of the membrane, which are essential for preserving sperm motility and facilitating key processes like capacitation and the acrosome reaction, both of which are vital for successful fertilization. Moreover, lipid peroxidation disrupts the acrosomal membrane, diminishing the sperm’s capacity to fuse with the oocyte. Consequently, oxidative damage induced by lipid peroxidation directly impairs sperm motility and fertilization potential, thereby contributing to infertility.

Oxidative stress also induces DNA fragmentation in spermatozoa, leading to the formation of single-strand and double-strand breaks in sperm DNA. This damage is exacerbated by the limited capacity of sperm to repair oxidative damage due to the compact packaging of DNA with protamines and the minimal availability of DNA repair mechanisms. DNA fragmentation reduces the genetic integrity of sperm, impairing fertilization and increasing the risk of poor reproductive outcomes, such as miscarriage, failed implantation, and the transmission of genetic defects to offspring. In cases where ART are used, the presence of fragmented sperm DNA can further complicate successful fertilization and increase the risk of developmental abnormalities in embryos. Protein oxidation is another critical molecular mechanism by which oxidative stress impairs sperm function. ROS can modify sperm proteins, leading to structural changes that impair motility, metabolic processes, and the sperm’s ability to bind to the oocyte. Oxidation of cytoskeletal proteins, such as actin and tubulin, compromises the structural integrity of the sperm’s flagellum, reducing motility and the sperm’s ability to reach the oocyte. Additionally, oxidative damage to metabolic enzymes reduces ATP production, further impairing sperm motility and energy availability. Protein oxidation also affects surface proteins involved in sperm-egg recognition, further diminishing fertilization potential.

The substantial impact of oxidative stress on sperm functionality and male fertility underscores the necessity for early detection and proactive management in infertile men. Prompt diagnosis of oxidative stress in seminal fluid is feasible through evaluating biomarkers such as levels of ROS, lipid peroxidation byproducts like MDA, and DNA fragmentation indices. Systematic screening for oxidative stress should be prioritized for men exhibiting infertility, especially those with contributing risk factors such as smoking, obesity, or exposure to environmental toxins. Such early identification is crucial for directing timely interventions that can alleviate the harmful effects of oxidative stress on spermatozoa and enhance fertility prospects. Management strategies for oxidative stress-induced sperm damage predominantly concentrate on boosting antioxidant defenses via dietary supplements and lifestyle adjustments. Antioxidant treatment, extensively examined for its potential to decrease ROS levels and shield sperm against oxidative harm, includes the use of supplements like vitamins C and E, coenzyme Q10, glutathione, selenium, and zinc. These antioxidants not only neutralize ROS and diminish lipid peroxidation but also safeguard sperm DNA from oxidative injuries. Furthermore, they bolster mitochondrial function, curb the generation of mitochondrial ROS, and augment sperm motility. Clinical research has validated the effectiveness of antioxidant supplements in mitigating oxidative stress, enhancing sperm quality, and elevating pregnancy rates among couples grappling with infertility.

In addition to antioxidant supplementation, lifestyle modifications are crucial in reducing oxidative stress and improving sperm health. Men are encouraged to adopt healthier habits, such as quitting smoking, reducing alcohol consumption, managing stress, and improving their diet. Smoking and excessive alcohol intake are well-known contributors to oxidative stress, depleting antioxidant reserves and increasing ROS production. Adopting a diet rich in antioxidants—such as fruits, vegetables, nuts, seeds, and omega-3 fatty acids—can provide additional protection against oxidative damage and enhance the body’s natural antioxidant defenses. Regular physical activity and stress management techniques, such as meditation and yoga, can also reduce oxidative stress and improve overall reproductive health. Despite the progress made in understanding and managing oxidative stress in male infertility, there remain several areas for future research. One of the primary challenges is optimizing antioxidant therapies. While antioxidant supplementation has shown promise, there is still a need to determine the optimal types, doses, and combinations of antioxidants that are most effective for reducing oxidative stress and improving fertility outcomes. Research should focus on personalized approaches to antioxidant therapy, taking into account individual variations in ROS levels, antioxidant capacity, and underlying health conditions that may influence the effectiveness of treatment.

Another area for future research is the development of novel diagnostic tools for detecting oxidative stress in semen. While current methods focus on assessing ROS levels, lipid peroxidation products, and DNA fragmentation, more advanced techniques are needed to provide a comprehensive assessment of oxidative damage in spermatozoa. Proteomics and metabolomics approaches could be used to identify specific oxidative modifications in sperm proteins and metabolites, providing more detailed insights into the molecular mechanisms of oxidative stress-induced sperm damage. These tools could also help identify new therapeutic targets for reducing oxidative stress and improving sperm function. Furthermore, research should explore the long-term impact of oxidative stress on male fertility and offspring health. While it is clear that oxidative stress impairs sperm function and increases the risk of infertility, there is growing evidence to suggest that oxidative damage to sperm DNA may have long-term consequences for offspring, including increased susceptibility to genetic disorders and developmental abnormalities. Investigating the mechanisms by which oxidative stress affects epigenetic modifications in sperm and how these modifications may influence embryonic development and offspring health is a promising area of research that could have significant implications for reproductive medicine.

In summary, oxidative stress plays a pivotal role in male infertility, primarily through its deleterious effects on spermatozoa, which manifest as diminished motility, reduced fertilization capabilities, and compromised genetic integrity. Prompt detection and proactive intervention are imperative for the effective management of oxidative stress in men suffering from infertility, with antioxidant supplementation and lifestyle modifications serving as the cornerstone of treatment strategies. Nonetheless, there remains a critical need for further research to refine antioxidant therapies, develop more sophisticated diagnostic tools, and elucidate the long-term effects of oxidative stress on male fertility and the health of subsequent generations. By surmounting these challenges, we can enhance the management of oxidative stress-induced male infertility and improve reproductive outcomes for affected couples.
